# Expression, regulation and function of phosphofructo-kinase/fructose-biphosphatases (PFKFBs) in glucocorticoid-induced apoptosis of acute lymphoblastic leukemia cells

**DOI:** 10.1186/1471-2407-10-638

**Published:** 2010-11-23

**Authors:** Michela Carlet, Kristina Janjetovic, Johannes Rainer, Stefan Schmidt, Renate Panzer-Grümayer, Georg Mann, Martina Prelog, Bernhard Meister, Christian Ploner, Reinhard Kofler

**Affiliations:** 1 Division Molecular Pathophysiology, Biocenter, Medical University of Innsbruck, Innsbruck, Austria; 2 Tyrolean Cancer Research Institute, Innsbruck, 6020-Austria; 3 Department of Hematology and Oncology, Medical University of Innsbruck, Innsbruck, Austria; 4 Children's Cancer Research Institute and St. Anna Kinderspital, Vienna, Austria; 5 Department of Pediatrics I, Medical University of Innsbruck, Innsbruck, Austria; 6 Department of Pediatrics II, Medical University of Innsbruck, Innsbruck, Austria; 7 Institute of Legal Medicine, Medical University Innsbruck, Innsbruck, Austria

## Abstract

**Background:**

Glucocorticoids (GCs) cause apoptosis and cell cycle arrest in lymphoid cells and constitute a central component in the therapy of lymphoid malignancies, most notably childhood acute lymphoblastic leukemia (ALL). PFKFB2 (6-phosphofructo-2-kinase/fructose-2,6-biphosphatase-2), a kinase controlling glucose metabolism, was identified by us previously as a GC response gene in expression profiling analyses performed in children with ALL during initial systemic GC mono-therapy. Since deregulation of glucose metabolism has been implicated in apoptosis induction, this gene and its relatives, PFKFB1, 3, and 4, were further analyzed.

**Methods:**

Gene expression analyses of isolated lymphoblasts were performed on Affymetrix HGU133 Plus 2.0 microarrays. GCRMA normalized microarray data were analyzed using R-Bioconductor packages version 2.5. Functional gene analyses of *PFKFB2-15A *and *-15B *isoforms were performed by conditional gene over-expression experiments in the GC-sensitive T-ALL model CCRF-CEM.

**Results:**

Expression analyses in additional ALL children, non-leukemic individuals and leukemic cell lines confirmed frequent *PFKFB2 *induction by GC in most systems sensitive to GC-induced apoptosis, particularly T-ALL cells. The 3 other family members, in contrast, were either absent or only weakly expressed (*PFKFB1 *and *4*) or not induced by GC (*PFKFB3*). Conditional PFKFB2 over-expression in the CCRF-CEM T-ALL *in vitro *model revealed that its 2 splice variants (PFKFB2-15A and PFKFB2-15B) had no detectable effect on cell survival. Moreover, neither PFKFB2 splice variant significantly affected sensitivity to, or kinetics of, GC-induced apoptosis.

**Conclusions:**

Our data suggest that, at least in the model system investigated, PFKFB2 is not an essential upstream regulator of the anti-leukemic effects of GC.

## Background

Glucocorticoid (GC)-induced apoptosis is a phenomenon of considerable physiologic and therapeutic significance. Physiologically, it has been implicated in the shaping of the immune repertoire and controlling immune responses, and therapeutically it has been exploited in the treatment of lymphoid malignancies, most notably childhood acute lymphoblastic leukemia (ALL), where good response to introductory hormone treatment predicts a favourable over-all outcome [1]. GCs mediate most of their effects via their cognate receptor (GR), a ligand-activated transcription factor of the large nuclear transcription factor family. GC-induced apoptosis critically depends on sufficient levels of GR and subsequent alteration in gene expression, but the precise nature of the GC-regulated genes responsible for the anti-leukemic GC effects remains elusive (for reviews see)[1-4].

To further address this issue, we previously exploited a comparative expression profiling strategy using "whole genome" microarrays (Affymetrix HGU133 plus 2.0) to delineate the GC response in primary lymphoblasts from children suffering from ALL as well as from several other biological systems of GC-sensitivity and resistance [5]. Only a small number of genes were regulated by GC in the majority of patients, which might reflect the heterogeneity of the disease. Among these, *PFKFB2 *(6-phosphofructo-2-kinase/fructose-2,6-biphosphatase-2), a key regulator of glycolysis [6], was one of the most frequently regulated genes. It was induced more than 4-fold in all 3 T-ALL cases as well as in the T-ALL cell line CCRF-CEM. More than 2-fold induction was observed in 8/10 children with precursor B-ALL and 1/1 adult with precursor B-ALL, but not in peripheral blood lymphocytes from 2 healthy donors treated with GC (such cells do not undergo apoptosis in response to GC) [5]. Interestingly, the second GC-sensitive ALL cell line tested in this study, 697/EU-3, and mouse thymocytes failed to regulate *PFKFB2*. In conclusion, *PFKFB2 *was induced in many primary ALL cells (particularly T-ALLs), but not in non-malignant lymphoid lineage cells, raising the possibility that this gene might play a functional role in GC-induced apoptosis in malignant lymphoid cells such as ALL.

PFKFB2 is a member of a small gene family encoding 4 PFKFB isoenzymes. PFKFBs regulate formation and degradation of fructose-2,6-biphosphate (F-2,6-P2), a signalling molecule that controls glycolysis by regulating phosphofructokinase-1 (PFK-1) activity [7,8]. PFKFBs function as bifunctional enzymes: either they catalyze the synthesis of F-2,6-P2, a potent allosteric effector of PFK-1, *via *their 6-phosphofructo-2-kinase activity, or they degrade F-2,6-P2 to fructose 6-phosphate by acting as a fructose-2,6-biphosphatase. The kinase to biphosphatase activity ratio (K:B) is determined by the expression of specific isoforms, post-translational modifications of the enzyme as well as by numerous catabolic metabolites such as α-glycerol phosphate, citrate or ATP/GTP levels in the cells [9]. Initially identified in rat hepatocytes [8], 4 mammalian PFKFB isoenzymes have been described in different tissues and developmental stages, i.e., PFKFB1 in liver, skeletal muscles and fetal tissues; PFKFB2 in heart, kidney, pancreas islets and lymphoid tissues; PFKFB3 ubiquitously, particularly in brain; and the testis-specific PFKFB4. All isoenzymes share a highly conserved core structure comprising the kinase and phosphatase activities. The isoenzymes and their isoforms mainly differ in their N- and C-terminal variable regions, however, all of them are active as homodimers where the kinase domains come together in a head-to-head fashion, whereas the phosphatase domains function as monomers [7].

The human *PFKFB2 *gene codes for 2 isoforms (15A and 15B) generated by alternative splicing of the terminal exon 15, thus differing in their C-terminal amino acid sequence [6]. Interestingly, in the more frequently investigated PFKFB2-15A isoform, this 54 amino acid region contains two PKA-sensitive phosphorylation sites [10], i.e. Ser466 and Ser483, which have been shown to play an important role in the regulation of kinase- and phosphatase activity. Thus, phosphorylation of Ser466 increased kinase affinity to fructose-6-phosphate (Fru-6-P), whereas dephosphorylation of the same residues entailed increased phosphatase activity [11]. Since the PFKFB2-15B isoform lacks the above kinase-activating phosphorylation sites, it might lack kinase activity, raising the possibility that the 2 isoforms may have opposite functions.

Recently, alterations in glucose metabolism have been implicated in cell death and survival decisions, particularly in the lymphoid lineage [12] and in transformed cells [13]. Thus, lymphocytes depend upon extracellular signals transmitted *via *surface receptors and so-called survival kinases, like PKB/Akt, to maintain their viability. Part of these survival signals impinge on glucose metabolism, e.g., by increasing the expression of glucose transporters [14], with the net outcome of increased glycolysis and ATP production. Decline in glycolytic activity and ATP/ADP ratios results in integrity loss of mitochondria, the central regulators of metabolism and survival [15], with subsequent Bax-Bak dependent cytochrome-C release and cell death. Similarly, tumor cells depend upon glycolytic flux (Warburg effect)[16,17], which provides them with building blocks required for growth and energy. Hence, interference with "tumor metabolism" has become a promising target for cancer therapy [13].

The crucial role of PFKFBs in controlling glycolysis, along with the fact that one member of this small gene family, *PFKFB2*, was frequently regulated by GC in malignant lymphoblasts from children suffering from childhood ALL [5], makes this enzyme an interesting candidate for the anti-leukemic effects of GC. To further address this issue, we investigated expression and GC regulation of all 4 known *PFKFB *family members in the previously published and additional childhood ALL patients and in additional *in vitro *leukemic systems. We found that only *PFKFB2 *showed consistent GC-induction, particularly, although not exclusively, in T-ALL systems. To assess a possible functional significance of this regulation, we performed conditional over-expression using the 2 known PFKFB2 splice variants in the T-ALL cell model CCRF-CEM in which both variants are dramatically induced by GC. However, neither splice variant replicated the apoptotic GC effects nor entailed over-expression a consistent modulating effect on GC-induced leukemia apoptosis.

## Methods

### Patients, cell lines and tissue culture

The ALL children enrolled in this study were admitted to the Department of Pediatrics, Innsbruck Medical University, or the St. Anna Kinderspital in Vienna from September to December 2009, and treated according to the BFM protocol 2000 (for protocol details see: http://boriskononenko.files.wordpress.com/2009/09/1-protokoll-all-bfm-2000-mit-amendments-version-26-09-07-d0bad0bed0bfd0b8d18f1.pdf). To analyze the GC response in non-leukemic peripheral blood lymphocytes, Ficoll-purified peripheral blood mononuclear cells from 2 healthy volunteers and 3 children with epilepsia treated with a single injection of GC were included. The study was approved by the Ethics Committees of the Innsbruck Medical University (EK1-1193-172/35 for ALL children and UN2821 for epileptic children) and the Kinderspital in Vienna, and written informed consent was obtained from the patients and/or parents or custodians. The characteristics of 3 children with T-ALL and 10 with precursor B-ALL have been published previously [5], the remaining ALL children (3 T-ALLs and 15 precursor B-ALLs) showed similar clinical findings [Rainer et al. in preparation]. All 3 patients with epilepsia (E3, a 14 year-old girl; E4, a 6 year-old boy; E5, an 11 year-old boy) had an idiopathic generalized epilepsia without any evidence of metabolic, endocrinological, immunological, hematological or infectious disease. The 6 year old girl (epilepsia diagnosed at 4 years of age) was on Valproinate and Clobazam, the 9 year old girl (epilepsia diagnosed at 8 year of age) on Valproinate and Rufinamide, and E5 (epilepsia diagnosed at 2 years of age) on Valproinate and Sultiam. Because of therapy-refractory epilepsia, the 3 patients received their first GC pulse therapy with dexamethasone (Fortecortin, Merck, Vienna, Austria) 20 mg/m^2 ^administered intravenously over 1 hour. Blood samples were taken at 2, 6 and 24 hours after drug administration.

The T-ALL cell lines CCRF-CEM-C7H2 [18], 6 GC-sensitive and 6 GC-resistant derivatives CCRF-CEM-C7H2 [19], CEM-C7H2-2C8 [20], a CEM-C7H2 derivative with constitutive expression of the tetracycline-regulated reverse transactivator, rtTA [21], MOLT4 (CRL-1582, ATCC, Rockville, MD), and Jurkat (untransfected and a rat GR-transfected derivative [22]), the precursor B-cell lines 697/EU-3 (ACC 42, DSMZ, Braunschweig, Germany), NALM6 (ACC 128, DSMZ), RS4;11 (ACC 508, DSMZ) and AT-1 [23], and Burkitt's lymphoma Daudi (CCL-213, ATCC) were cultured in RPMI 1640 supplemented with 10% fetal calf serum and 2 mM L-glutamine at 37°C, 5% carbon-dioxide and saturated humidity. HEK293T packaging cells (ATCC, Manassas, VA) were cultured in DMEM supplemented as above. The cells were free of mycoplasma infection and their authenticity was verified by DNA fingerprinting, as detailed previously [24]. Doxycycline was dissolved (100 μg/ml) in phosphate-buffered saline and dexamethasone (10^-4^M) in 100% ethanol. The final ethanol-concentration in the dexamethasone-treated and control cultures was maintained at 0.1%. All above reagents were from Sigma (Vienna, Austria).

### Expression profiling

Our procedure for generating mRNA expression profiles on Affymetrix HGU133 plus 2.0 microarrays has been detailed previously [5]. Briefly, RNA from peripheral blood lymphoblasts of patients prior to, or at 6-8 or 24 hours after initiation of systemic GC monotherapy (following the BFM therapy protocol recommendations), from healthy volunteers or epileptic children 6 hours after GC treatment, and GC-sensitive or GC-resistant cell lines, was converted into labeled target and hybridized to Affymetrix HGU133 plus 2.0 microarrays according to standard protocols. The microarray data from the 31 ALL-patients and 5 non-leukemic individuals were normalized separately from those of the 12 cell lines using GCRMA [25] after positive evaluation of comprehensive raw data quality assessment. The analysis was performed in R using packages from Bioconductor [26] version 2.5. Annotation of probe sets to genes based on Affymetrix NetAffx annotation database version 22 and location of probe sets relative to the 3' end of the transcripts were manually inspected using the Ensembl genome browser for genes *PFKFB1*, *PFKFB2*, *PFKFB3 *and *PFKFB4*. Microarray data for the samples of the 13 ALL children and 2 healthy donors were published previously [5] and are available at NCBI's GEO database (series GSE2677 and GSE2842). Microarray data for the 12 cell lines (GSE22152) and 4 additional non-leukemic individuals (GSE22779) have been deposited at GEO.

### Apoptosis determinations

Apoptosis was determined by FACS analysis of propidium iodide (PI)-treated permeabilized cells [27] as previously detailed [28]. Briefly, cells were analyzed with a FACScan cytometer (Becton Dickinson Biosciences, San Jose, CA) in combination with CellQuest Pro software (Becton Dickinson Biosciences) acquiring forward scatter/sideward scatter, FL-2 H (log), and FL-3 H (linear). In FL-2 H, the percentage of nuclei with reduced DNA-content (SubG1 peak) was assessed.

### Immunoblotting

Our immunoblotting procedure has been detailed recently [29]. Briefly, proteins were extracted from 5 × 10^6 ^cells in 100 μl RIPA-buffer, quantified by Bradford analysis, mixed with 40 μl loading buffer (4 × SSB, 5% β-mercaptoethanol), denatured, fractionated on a 12.5% SDS-PAGE and electroblotted onto nitrocellulose. The membranes were incubated overnight with rabbit polyclonal antibodies against PFKFB2 (N-term, AP8146a, Abgent), PFKFB2 phosphorylated Ser466 (University of Dundee, UK) or mouse monoclonal antibodies against α-TUBULIN (DM1A, CalBiochem, Nottingham, UK) as a loading control. Specifically bound antibodies were detected with anti-rabbit, anti-sheep or anti-mouse horseradish-peroxidase-conjugated secondary antibodies (Amersham Pharmacia Biotech, Uppsala, Sweden) and visualized by chemiluminescence (ECL, Amersham) and subsequent exposure to AGFA Curix X-ray films for 1 second to 30 minutes.

### Generation of CCRF-CEM derivatives with doxycycline-induced PFKFB2-15A and -15B expression

The lentiviral conditional expression constructs pHR-tetCMV-PFKFB2-15A and -15B (U264 and U265) were generated using the GATEWAY™technology (Invitrogen, Carlsbad, CA). The details of this procedure and the generation of stable clonal cell lines with tetracycline-regulated expression of cDNAs cloned into such constructs has been described previously [30]. In brief, human sequences coding for *PFKFB2-15A *and *-15B *mRNA were PCR-amplified using unique SacI-flanked forward primer 5'-GAGCTCTGTGCTCGACGAGCTCGT-3' and XbaI-flanked isoform specific reverse primers 5'-TCTAGATGGGTCTTCGGCTAGT-3' (for PFKFB2-15A) and 5'-TCTAGAGTAGATCCCAGTCGT-3' (for PFKFB2-15B) and cDNA from CEM-C7H2 cells as template. The purified PCR product was cloned into the SacI-XbaI site of pENTR-MCS-deltaNcoI (U243), thereby generating pENTR207-PFKFB2-15A (U260) and pENTR207-PFKFB2-15B (U263). Constructs were sequence-verified and subsequently recombined into the "destination vector" pHR-tetCMV-Dest-IRES-GFP (U192) to generate pHR-tetCMV-PFKFB2-15A-ires-GFP (U264) and pHR-tetCMV-PFKFB2-15B-ires-GFP (U265), as previously described [30]. The lentiviral plasmids were transfected into 293T packaging cells together with pVSV-G and pSPAX (kindly provided by Didier Trono), and the lentivirus-containing supernatants were used to transduce CEM-C7H2-2C8, which constitutively express rtTA [20]. After limiting dilution cloning, three clonal cell lines expressing the PFKFB2-15A isoform, termed CEM-PFKFB2-15A#C3, #D6 and #E8, and three expressing PFKFB2-15B named CEM-PFKFB2-15B#65, #66 and #95, were selected for further experiments.

### Real time RT-PCR

For high through-put real time RT-PCR, 50 μl of diluted cDNA (2 ng/μl) were added to 50 μl of TaqMan Universal MasterMix (Applied Biosystems, Foster City, CA) and introduced into microfluidic cards containing real time RT-PCR mixes for human *PFKFB2 *(Hs01015408_m1, Applied Biosystems) and TATA box-binding protein for mRNA normalization (*TBP*, Hs00427620_m1) according to the manufacturer's guidelines. After equilibration at RT, the channels were filled with 100 μl of reaction mix and centrifuged two times for 1 minute at 1000 rpm. Thereafter, the cards were sealed, loaded into the HT7900 real time machine (Applied Biosystems), and run with a 2-step PCR thermo-protocol that included an initial 94.5°C step for 10 min followed by 40 cycles of 97°C for 15 sec alternating with 60°C for 1 min. Fluorescence signal intensities were read during the 60°C temperature step. Similarly, but on conventional 96 well plates, mRNA encoding the 2 *PFKFB2 *splice variants 15A and 15B were quantified (Hs01015410_m1 and Hs01016554_m1, Applied Biosystems, for PFKFB2-15A and PFKFB2-15B, respectively). Primary real-time PCR data analysis was performed with SDS software version 2.2.1, and further analysis was performed in R (version 2.8). Data from 3 technically replicated measurements were averaged and normalized to the internal *TBP *control. Log2 fold change values (M values) were calculated for 3 biological replicates by comparing normalized real-time PCR data from GC-treated samples against data from the corresponding control samples. M values were averaged for the 3 biological replicates and p-values were calculated (Student's t-test) to test against the null hypothesis of no differential expression (mean M = 0).

## Results

### Expression and regulation of *PFKFB1*, *3 *and *4 *isoenzymes in lymphocytes and malignant lymphoblasts

In a previous study, we showed that *PFKFB2 *is regulated by GC in 11/13 children suffering from T-ALL and preB-ALL [5]. To elucidate whether GC exclusively regulated *PFKFB2 *in lymphoid cells, or whether other *PFKFB*-isoenzymes are susceptible to GC-treatment as well, we re-analyzed this data using the more robust GCRMA normalization and included 18 additional ALL patients undergoing GC therapy as well as 3 non-leukemic children who received GC as part of their epilepsia treatment, and 6 GC-sensitive and 6 GC-resistant derivatives of the CEM-C7H2 T-ALL cell line. Additional file 1, Table S3 summarizes mean expression levels (mE-value) and mean GC regulation (mM-value) of the 3 isoforms, i.e. *PFKFB1*, *PFKFB3 *and *PFKFB4*, in these samples, organized in the following groups: T-ALLs, precursor B-ALLs [as one group and as 3 separate groups based on molecular defects: hyperdiploid, *ETV6/RUNX1 *(previously *TEL/AML1*) translocation, and a heterogeneous group termed "others"], non-leukemic donors and GC-sensitive and -resistant CCRF-CEM derivates. Data for individual patients and cell lines are depicted in Additional file 1, Tables S1 and S2. *PFKFB1 *and *PFKFB4 *isoenzymes were neither detectably expressed in any of the investigated systems nor regulated by GC. The brain/placenta- and tumor-specific *PFKFB3 *isoenzyme showed expression levels comparable to those of *PFKFB2 *(for comparisons see Table 1), however, in contrast to *PFKFB2*, its expression was not upregulated by GC. Interestingly, expression of *PFKFB3 *was also observed in peripheral blood lymphocytes from non-leukemic donors, indicating that this isoenzyme is not restricted to placenta and brain or malignant tissues. In conclusion, the *PFKFB1 *and *4 *isoenzymes were neither expressed nor regulated by GC and, hence, might not play a role in the anti-leukemic GC effects. *PFKFB3 *is expressed, but shows no significant GC-regulation, excluding it from the list of likely candidate genes.

**Table 1:** Expression and regulation of PFKFB2 in different lymphoid systems1

	Expression (mE-value)		Regulation (mM-value)		Regulation (mM-value)	
**samples**	**15A**	**15B**	**15A 6/8 h**	**15A 24 h**	**15B 6/8 h**	**15B 24 h**

**T-ALL (6)**	6.3 ± 1.5	4.0 ± 1.9	1.7 ± 1.5	2.4 ± 1.4	1.9 ± 2.3	2.7 ± 2.3
**Precursor B-ALL (25)**	4.6 ± 2.7	3.0 ± 1.7	0.7 ± 0.8	1.1 ± 1.2	0.6 ± 0.9	0.9 ± 1.1
- **hyperdiploid (7)**	2.1 ± 0.7	1.5 ± 0.2	0.7 ± 0.6	0.9 ± 1.1	0.3 ± 0.4	0.3 ± 0.3
- **EVT6/RUNX1 (10)**	6.7 ± 1.7	4.4 ± 1.5	0.5 ± 1.1	0.8 ± 1.5	0.8 ± 1.1	1.1 ± 1.2
**- others (8)**	4.2 ± 2.7	2.4 ± 1.3	0.9 ± 0.8	1.5 ± 0.8	0.6 ± 0.8	1.3 ± 1.2
Non-leukemic donors (6*)	3.4 ± 0.2	2.0 ± 0.4	0.5 ± 0.7	ND	0.2 ± 0.3	ND
**CEM-C7H2-S-lines (6)**	2.3 ± 0.1	1.8 ± 0.1	3.1 ± 0.9	ND	1.8 ± 0.4	ND
**CEM-C7H2-R-lines (6)**	2.7 ± 0.3	1.8 ± 0.1	2.0 ± 2.0	ND	1.6 ± 1.3	ND

^1^Expression and regulation of *PFKFB2 *in ChALL patients, non-leukemic controls (adult healthy donors and epileptic children) and sensitive (S) and resistant (R) CEM-C7H2 cell lines. Basal mean expression, before GC-treatment, is reported as mE-value ± SD; mean regulation was assessed after 6 and 24 hours and is expressed as mM-value ± SD. The total number of cases is reported in brackets next to the subgroup name. Data were assessed by analysis of Affymetrix HGU133 plus 2.0 microarrays. Abbreviation: ND, not determined.

### Expression and regulation of *PFKFB2-15A *and -*15B *in lymphocytes and malignant lymphoblasts

Next, we analyzed expression and GC regulation of the 2 splice variants of *PFKFB2 *in the above biological systems. As summarized in Table 1, basal expression of both *PFKFB2 *isoforms was variable: almost all T-ALLs (known to show particularly strong response to GC) and *ETV6/RUNX1 *positive ALLs (which have a good prognosis) showed intermediate, all other subgroups, no or very low expression. In all groups with detectable expression of *PFKFB2*, mRNA levels (as measured by signal intensity) of *PFKFB2-15A *were ~2-fold higher than those of *PFKFB2-15B*. Concerning regulation, T-ALL patients showed upregulation of both splice variants at both time points, with mean M values between 1.7 and 2.7. This was also true for precursor B-ALL patients, although the extent of regulation was considerably less (mean M values between 0.6 and 1.1).

On an individual basis (Additional file 1, Table S1), the T-ALL group appeared quite homogeneous, with 5/6 T-ALL children showing induction (as defined by an M-value >1, i.e., >2-fold) after 6 and/or 24 hours of GC-treatment. In hyperdiploid precursor B-ALL children, isoform *PFKFB2-15A *was upregulated in 5/7 cases, with one child presenting a tendency for downregulation (M-value = -0.6), while *PFKFB2-15B *induction was present only in 1 of the 7 children after 6 hours. The subgroup of children with the *ETV6/RUNX1 *translocation showed induction of *PFKFB2-15A *in 4/10 children, 2 showed a slight downregulation, and 4 lacked *PFKFB2-15A *regulation. A more homogeneous situation was observed for the *PFKFB2*-15B isoform, where induction was detected in 8/10 children, with the remaining 2 showing a tendency to downregulate *PFKFB2-15B *after the first time point. The last subgroup [low hyperdiploid, *E2A/PBX1 *translocation, t(8;4) and no chromosomal abnormalities] showed upregulated *PFKFB2-15A *in 7/8 cases and *PFKFB2-15B *in 4/8, and no regulation in the others. In contrast to the ALL samples where *PFKFB2 *induction was a frequent event, this was seen only once and only for *PFKFB2-15A *in the non-leukemic donors. Concerning the *in vitro *cell lines systems, all 6 GC-sensitive CEM-C7H2 subclones showed clear induction of *PFKFB2-15A *and *-15B*, with M values ranging from 1.6 to 4.0 (15A) and 1.1 to 2.3 (15B). The 6 R-lines resulted in a remarkably heterogeneous picture, with M-values ranging from 0.0 to 5.1 (15A) and 0.5 to 3.5 (15B). Thus, despite the complexity of the expression profiling data, *PFKFB2 *induction appeared to be a frequent feature in the GC response of T-lineage ALL and also occurred in precursor B-ALL, albeit with lower frequency. Moreover, *PFKFB2 *induction was absent or reduced in systems resistant to GC-induced apoptosis (non-leukemic donors, CEM-C7H2-R-lines).

### Expression and regulation of *PFKFB2 *isoforms in additional leukemic cell line models

**Figure 1:**
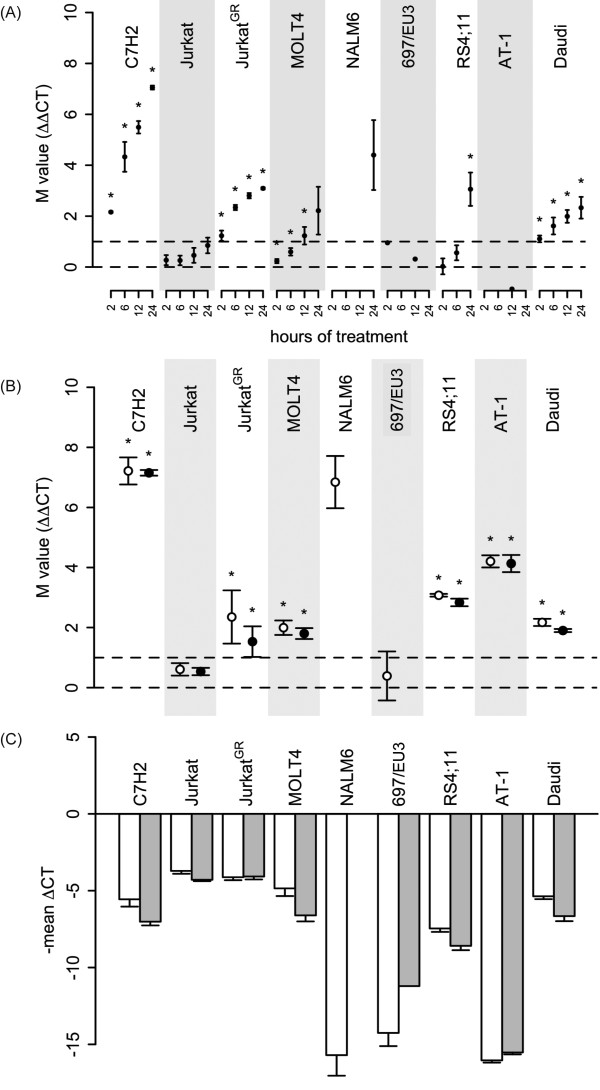
**Expression/regulation of *PFKFB2 *and its splice variants in leukemic cell lines**. (A) The cell lines were cultured as indicated in the presence of 10^-7^M dexamethasone or 0.1% ethanol as vehicle control, and analyzed for mRNA expression of *PFKFB2 *(using a primer pair recognizing both splice variants) and *TBP *as control by quantitative RT-PCR on microfluidic cards. *TBP*-normalized *PFKFB2 *expression levels (ΔC_T_) were used to determine GC regulation (ΔΔC_T_) expressed as mean M-values ± standard deviation. Asterisks indicate p-values of <0.05. *PFKFB2 *was undetectable in NALM6 (at 2, 6 and 12 hours) and AT-1 cells prior to, but clearly detectable, after GC treatment. Thus, the extent of regulation (ΔΔC_T_) could not be quantified. Asterisks indicate statistically significant changes of expression (p < 0.05) as calculated by a paired t-test from 3 replicated values. Regulations were considered biologically significant if the extent of differential expression was larger than 2-fold (indicated by the dashed horizontal line M = 1). (B) Regulation of *PFKFB2 *variants (open circles for *PFKFB2-15A*, black circles for *PFKFB2-15B*) after 24 hour exposure to 10^-7^M dexamethasone is expressed as mean M-value (ΔΔCT) ±SD. Asterisks indicate p < 0.05. In NALM6 cells, *PFKFB2-15B *was induced by GC as well. However, since the expression level in the ethanol-treated control sample was below the limit of detection, it was not possible to derive an M-value. In the 697/EU-3 cells, *PFKFB-15B *was undetectable after 24 hour GC exposure, which might be explained by the fact that *PFKFB2 *levels in 697/EU-3 were at the limit of detection of this assay or suggest a weak down-regulation. (C) Basal expression of the *PFKFB2-15A *(white bars) and -*15B *(grey bars) splice variants was detected in the same cell lines using specific Taqman quantitative RT-PCR and normalized to *TBP*. Expression levels are reported as minus mean ΔCT (ΔCT, CT values obtained with *TBP *primers minus CT values obtained with *PFKFB2-15A *or *-15B *primers, respectively). In AT-1, both splice variants, and in NALM6 the *PFKFB2-15A *variant, could be detected in the absence of dexamethasone, although at very low levels (presumably due to slightly higher efficiency of these RT-PCRs compared to the RT-PCR for both variants).

To further address whether *PFKFB2 *regulation was related to GC-sensitivity and/or preB-ALL and T-ALL origin, we treated 9 leukemia cell lines with 10^-7^M dexamethasone for 2, 6, 12 and 24 hours in biological triplicates and performed real time RT-PCR using primers covering the exon-3/exon-4 boundary present in both splice variants (Figure 1A). To assess the contribution of the individual splice variants, the 24 hour samples were further analyzed using primers specific for *PFKFB2-15A *and *-15B *(Figure 1B and 1C). As we previously reported [31], the extent of GC sensitivity in these cell lines was markedly different, i.e., untransfected Jurkat and MOLT4 T-ALL cell lines as well as AT-1 precursor B-ALL and Daudi Burkitt lymphoma cells were resistant to GC-induced apoptosis, while all others were GC-sensitive, although with varying kinetics (Additional file 1, Figure S1). Effects of GC on cell cycle progression were also determined [31] (Additional file 1, Figure S1) and showed that GC-induced apoptosis was frequently preceded or accompanied by an increase of cells in the G1 phase of the cell division cycle, whereas the cell lines resistant to GC-induced apoptosis were also resistant to the GC effects on the cell cycle. The notable exception was AT-1, in which G1 cell cycle arrest was observed in the complete absence of apoptosis. As shown in Figure 1A, *PFKFB2 *induction was a frequent event in these cell lines starting as soon as 2 hours after initial GC exposure and increasing up to 24 hours. In those instances where *PFKFB2 *induction was observed, both splice variants appeared to be similarly regulated (Figure 1B). There was no apparent correlation between *PFKFB2 *induction and GC sensitivity or T/B-lineage origin of the leukemic cell lines. Concerning basal levels, the 2 splice variants showed co-expression across the panel of leukemia cell lines (Figure 1C), with *PFKFB2-15A *levels slightly exceeding those of *-15B*, thus resembling the findings on the microarrays. In general, B-lineage cells revealed lower expression levels than T-lineage cells (again resembling the situation in patients), but there was no apparent correlation between basal *PFKFB2 *levels and GC-sensitivity. For instance, GC-sensitive C7H2 T-ALL cells had similar *PFKFB2 *levels as GC-resistant MOLT4 T-ALL. The same was true for GC-sensitive Jurkat^GR ^and their parental GC-resistant Jurkat cell line.

Similar to the situation in patients, GC-dependent induction was more pronounced in T-ALL cells than in B-lineage leukemias, but there was no obvious correlation with GC sensitivity across all samples. In the Jurkat T-ALL system, however, induction of *PFKFB2 *correlated with GC sensitivity, as we have similarly seen in the CCRF-CEM system using microarray technology. Although we were unable to definitively prove regulation at the protein level (for reasons explained in Additional file 1), the combined clinical and experimental mRNA data were compatible with the notion that induction of *PFKFB2 *contributes to GC-induced apoptosis, particularly in T-ALL cells.

### Effect of conditional over-expression of PFKFB2 isoforms on cell viability and GC-sensitivity

**Figure 2:**
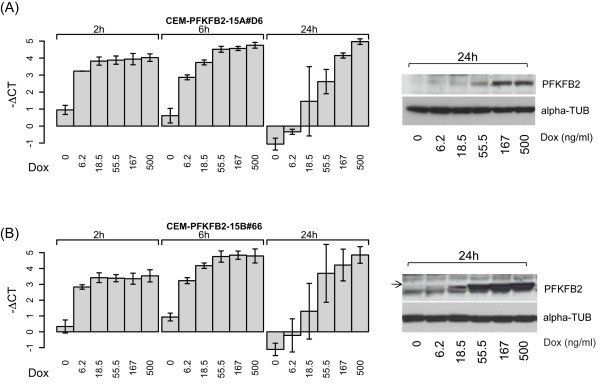
**Characterization of T-ALL cell lines with conditional PFKFB2-15A and -15B expression**. CEM-PFKFB2-15A #D6 (Figure 2A) and CEM-PFKFB2-15B #66 (Figure 2B) cells expressing PFKFB2-15A and -15B in a doxycycline-dependent manner, respectively, were cultured in the presence of increasing concentration of doxycycline (Dox) for the indicated time and analysed for PFKFB2 expression (see Figures S2 and S3 for the corresponding data for the remaining 4 cell lines used in this study). mRNA expression was assessed by using quantitative real time RT-PCR targeting the exons-3/exon-4 boundary present in both splice variants. The data derive from 3 biological replicates analyzed in triplicate and expressed as -ΔCT (i.e., CT value of *PFKFB2-15A *minus CT value of *TBP*). PFKFB2 protein was detected by immunoblotting with antibodies against PFKFB2 and α-TUBULIN as loading control (right panels). Shown are representative examples of cells treated with the indicated amounts of doxycycline for 24 hours.

**Figure 3:**
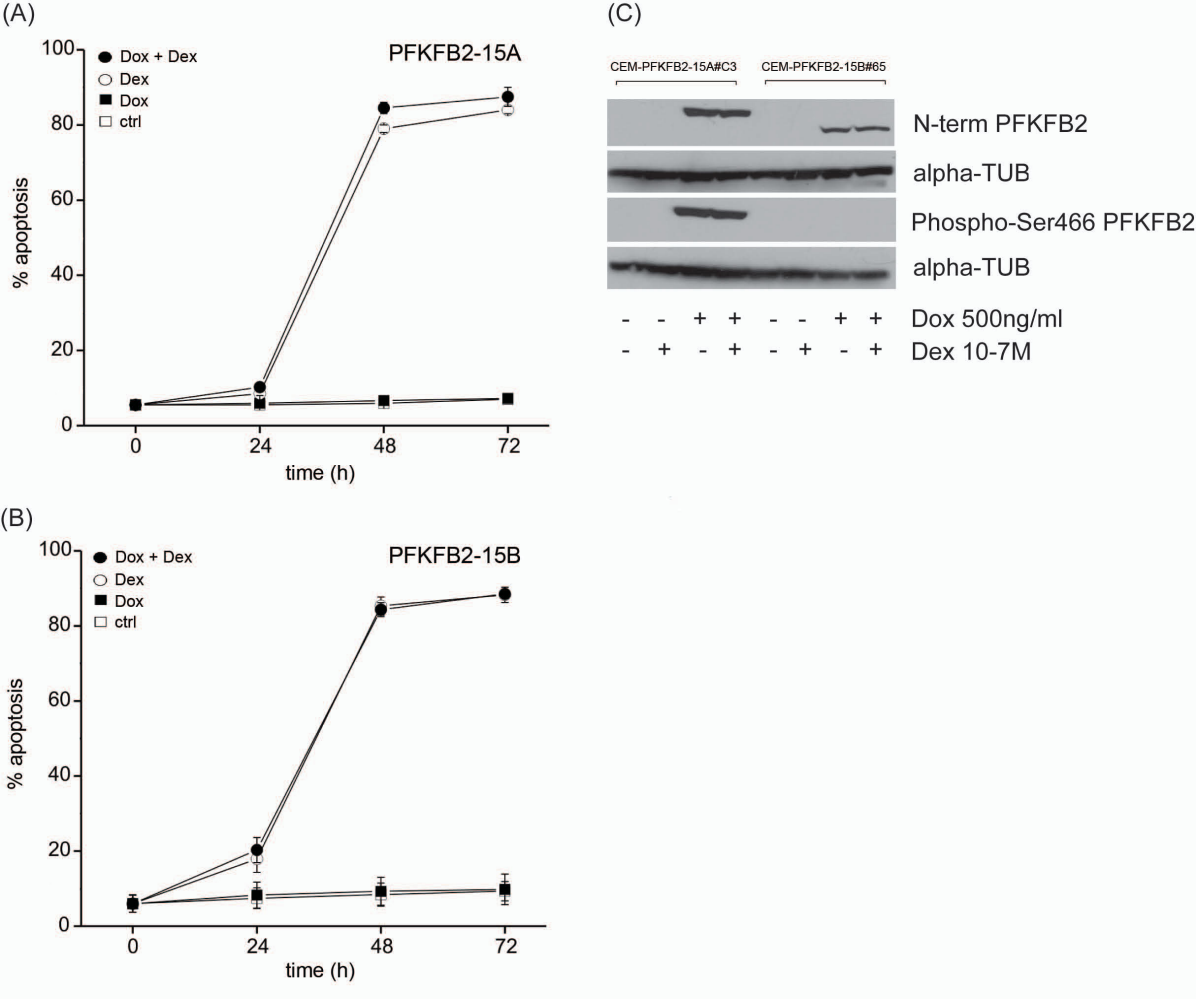
**Conditional PFKFB2 over-expression in CCRF-CEM T-ALL cells has no effect on GC-induced apoptosis**. CEM-PFKFB2-15A#C3, #D6 and #E8 cells (expressing PFKFB2-15A in a doxycycline-dependent manner, Figure 3A) and CEM-PFKFB2-15A#65, #66 and #95 cells (expressing PFKFB2-15B in a doxycycline-dependent manner, Figure 3B) were cultured in the absence of doxycycline and dexamethasone (open square), in the presence of 10^-7 ^M dexamethasone (open circle) or 500 ng/ml doxycycline (closed square), and with the combination of both (closed circle) for the indicated time and subjected to apoptosis determination using flow cytometric analyses of propidium iodide-stained nuclei ("apoptosis" refers to percent of events in the SubG1 window). Since all 3 cell lines for each isoform gave similar results, the data were pooled. Shown are the mean values±SD of specific apoptosis derived from biological triplicates of the 3 cell lines (i.e., 9 measurements per time point and treatment). Figure 3C: PFKFB2-15A and -15B protein expression was analyzed in the indicated cell lines with and without 24 h doxyxcyline induction (Dox, 500 ng/ml) and in the absence and presence of dexamethasone (Dex, 10^-7^M) by immunoblotting using an antibody against the shared N-terminal region (top panel) or Ser466 phosphorylated PFKFB2-15A (3^rd ^panel) with α-TUBULIN serving as loading control.

To evaluate a possible contribution of increased PFKFB2 levels to the anti-leukemic effects of GC, we generated cell lines conditionally overexpressing either PFKFB2-15A or -15B in a doxycycline-dependent manner. Three clones for each isoform (termed CEM-PFKFB2-15A#C3, #D6, #E8 and CEM-PFKFB2-15B#65, #66, #95, respectively) were analyzed for regulation of the transgene by quantitative RT-PCR and immunoblotting (Figure 2 and Additional file 1, Figure S2 and S3). In all instances we obtained doxycycline dose- and time-dependent induction of the PFKFB2 splice variants to levels as high as, or even exceeding, those obtained after GC treatment. Nevertheless, no effect was observed on cell viability, suggesting that induction of either PFKFB2 splice variant is not sufficient to explain apoptosis seen after GC exposure (Figure 3). In addition, the FACS data of PI-stained nuclei strongly suggested that there was no effect on cell cycle progression or GC-induced cell cycle arrest (data not shown). To investigate whether transgenic expression of PFKFB2 resulted in altered GC susceptibility, we treated the above T-ALL cell lines with 10^-7^M dexamethasone in the presence and absence of doxycycline (i.e., with and without transgenic PFKFB2 induction) and analyzed the extent of apoptosis (as measured by FACS analysis of propidium iodide incorporation) at various time points (Figure 3). Neither PFKFB2-15A nor -15B over-expression significantly changed GC sensitivity in these cells, although both isoforms were clearly detectable by immunoblotting (Figure 3C). Moreover, in the case of PFKFB2-15A where corresponding analyses can be performed, we observed that the activation-specific Ser466 [11] was phosphorylated, suggesting that the transgenic protein was not only sufficiently well expressed, but also active as a kinase. In conclusion, the data show that, at least in the investigated model system, neither PFKFB2-15A nor -15B over-expression mimics the anti-leukemic effects of GC, nor does it alter GC sensitivity.

## Discussion

In this study, we addressed the possible functional significance of GC-dependent *PFKFB2 *regulation in primary malignant lymphoblasts and leukemic cell line models. *PFKFB2 *was identified by microarray analysis of lymphoblasts isolated from GC-treated children suffering from ALL [5] as one of the most promising candidate genes, since it was regulated in the majority of patients and its deregulation should entail disturbances in glucose metabolism which, in turn, have been implicated in cell death induction in general [12,13]. More specifically, GC resistance was associated with increased glucose consumption [32], and interference with this glucose dependency by simultaneous addition of 2-deoxy-D-glucose (2-DG) increased *in vitro *sensitivity to GC [32,33]. We therefore hypothesized that the apoptotic effect of GC might be triggered, at least in part, by reducing the glycolytic activity of the cell, and that this GC effect is mediated by upregulation of *PFKFB2*.

To further test this hypothesis, we first performed detailed expression and regulation analysis of the 2 splice variants of this gene and of the 3 other members of the *PFKFB *gene family in several GC-sensitive and -resistant leukemic T-ALL and preB-ALL cell lines and peripheral blood cells from non-leukemic individuals. This made a possible contribution of the 3 other *PFKFB *isoenzymes to GC effects in such cells highly unlikely: *PFKFB1 *and -*4 *were not, or only very moderately, expressed and were not regulated by GC treatment, *PFKFB3 *was expressed, but not significantly regulated by GC (Additional file 1, Table S3). Concerning *PFKFB2*, we observed that both splice variants were expressed at similar levels (with *PFKFB2-15A *somewhat higher than -*15B*) and were co-regulated in most systems. Although the number of patients is limited, GC induction was more pronounced (both in terms of frequency and extent of regulation) in T-ALLs compared to precursor B-ALLs. Concerning correlation with GC sensitivity, all ALL children were considered GC-sensitive because all showed a reduction in blast counts within the first 24 to 48 hours of treatment. Three children (24, 699, 724) were classified as prednisolone poor responders based on a blast count of >1000/μl on day 8, yet they also showed GC-induction of at least 1 of the variants at 1 or both time points. GC-resistant cell lines and non-leukemic individuals (whose peripheral blood lymphocytes are considered resistant to GC-induced apoptosis)[34,35], clearly showed less GC-induction of *PFKFB2*. Thus, even though the correlation of GC-induction of *PFKFB2 *and cell death was not complete, the extended expression/regulation analyses further supported a possible role of PFKFB2 in GC-induced apoptosis, particularly in T-ALL cells.

We therefore performed conditional over-expression of both PFKFB2 splice variants in a GC-sensitive T-ALL model system, but these experiments showed no detectable effect of PFKFB2-15A or -15B on cell death, nor did their over-expression change sensitivity to, or kinetics of, GC-induced apoptosis. While these data clearly showed that induction of neither of the 2 splice variants suffices to elicit the anti-leukemic GC effects, further work is needed to dissect whether the effects of GC on glucose metabolism are mediated by induction of PFKFB2 (as we hypothesized based on the reported role of PFKFB enzymes) or whether other, perhaps less obvious, pathways are involved. Preliminary experiments addressing this issue suggested that, surprisingly, over-expression of both PFKFB2 splice variants seems to have little, if any, effect on lactate and ATP production, two indicators of glycolytic activity that are reduced after GC treatment. Thus, the current data show that induction of the 2 splice variants of PFKFB2 cannot explain the anti-leukemic GC effects. Whether alterations of glucose metabolism by other GC-dependent mechanisms might contribute to GC-induced leukemia apoptosis requires further investigations.

## Conclusions

Our study demonstrated that the heart-specific PFKFB2 isoenzyme is expressed and specifically induced by GC in malignant lymphoid cells, however, functional analysis of this gene in the human T-ALL cell line model CCRF-CEM revealed that its over-expression does not explain the anti-leukemic effects of GC.

## Competing interests

The authors declare that they have no competing interests.

## Authors' contributions

MC participated in most experiments and contributed to the writing of the manuscript. KJ generated the PFKFB2 constructs, JR performed the bioinformatic data analyses, SS, RP, GM, MP and BM provided clinical samples and corresponding data, CP coordinated the writing of the manuscript, and RK coordinated the entire study and contributed to the writing of the manuscript. The final version was seen and approved by all authors.

## Pre-publication history

The pre-publication history for this paper can be accessed here:

http://www.biomedcentral.com/1471-2407/10/638/prepub

## Supplementary Material

Additional file 1 **Supplement**. Supplementary information consisting of 3 Tables, 4 Figures and additional discussion section. Click here for file

## References

[B1] Schmidt S Rainer J Ploner C Presul E Riml S Kofler R Glucocorticoid-induced apoptosis and glucocorticoid resistance: Molecular mechanisms and clinical relevance Cell Death Differ 2004 11 Suppl 1 S45 S55 10.1038/sj.cdd.4401456 15243581

[B2] Distelhorst CW Recent insights into the mechanism of glucocorticosteroid-induced apoptosis Cell Death Differ 2002 9 6 19 10.1038/sj.cdd.4400969 11803370

[B3] Gross KL Lu NZ Cidlowski JA Molecular mechanisms regulating glucocorticoid sensitivity and resistance Mol Cell Endocrinol 2009 300 7 16 10.1016/j.mce.2008.10.001 19000736 PMC2674248

[B4] Tissing WJ Meijerink JP den Boer ML Pieters R Molecular determinants of glucocorticoid sensitivity and resistance in acute lymphoblastic leukemia Leukemia 2003 17 17 25 10.1038/sj.leu.2402733 12529655

[B5] Schmidt S Rainer J Riml S Ploner C Jesacher S Achmüller C Presul E Skvortsov S Crazzolara R Fiegl M Raivio T Jänne OA Geley S Meister B Kofler R Identification of glucocorticoid response genes in children with acute lymphoblastic leukemia Blood 2006 107 2061 2069 10.1182/blood-2005-07-2853 16293608

[B6] Heine-Suner D Diaz-Guillen MA Lange AJ Rodriguez dC Sequence and structure of the human 6-phosphofructo-2-kinase/fructose-2,6-bisphosphatase heart isoform gene (PFKFB2) Eur J Biochem 1998 254 103 110 10.1046/j.1432-1327.1998.2540103.x 9652401

[B7] Rider MH Bertrand L Vertommen D Michels PA Rousseau GG Hue L 6-phosphofructo-2-kinase/fructose-2,6-bisphosphatase: head-to-head with a bifunctional enzyme that controls glycolysis Biochem J 2004 381 561 579 10.1042/BJ20040752 15170386 PMC1133864

[B8] Colosia AD Lively M El-Maghrabi MR Pilkis SJ Isolation of a cDNA clone for rat liver 6-phosphofructo 2-kinase/fructose 2,6-bisphosphatase Biochem Biophys Res Commun 1987 143 1092 1098 10.1016/0006-291X(87)90364-0 3032183

[B9] Okar DA Manzano A Navarro-Sabate A Riera L Bartrons R Lange AJ PFK-2/FBPase-2: maker and breaker of the essential biofactor fructose-2,6-bisphosphate Trends Biochem Sci 2001 26 30 35 10.1016/S0968-0004(00)01699-6 11165514

[B10] Kitamura K Kangawa K Matsuo H Uyeda K Phosphorylation of myocardial fructose-6-phosphate,2-kinase: fructose-2,6-bisphosphatase by cAMP-dependent protein kinase and protein kinase C. Activation by phosphorylation and amino acid sequences of the phosphorylation sites J Biol Chem 1988 263 16796 16801 2846551

[B11] Bertrand L Alessi DR Deprez J Deak M Viaene E Rider MH Hue L Heart 6-phosphofructo-2-kinase activation by insulin results from Ser-466 and Ser-483 phosphorylation and requires 3-phosphoinositide-dependent kinase-1, but not protein kinase B J Biol Chem 1999 274 30927 30933 10.1074/jbc.274.43.30927 10521487

[B12] Plas DR Rathmell JC Thompson CB Homeostatic control of lymphocyte survival: potential origins and implications Nat Immunol 2002 3 515 521 10.1038/ni0602-515 12032565

[B13] Tennant DA Duran RV Gottlieb E Targeting metabolic transformation for cancer therapy Nat Rev Cancer 2010 10 267 277 10.1038/nrc2817 20300106

[B14] Rathmell JC Vander Heiden MG Harris MH Frauwirth KA Thompson CB In the absence of extrinsic signals, nutrient utilization by lymphocytes is insufficient to maintain either cell size or viability Mol Cell 2000 6 683 692 10.1016/S1097-2765(00)00066-6 11030347

[B15] Hammerman PS Fox CJ Thompson CB Beginnings of a signal-transduction pathway for bioenergetic control of cell survival Trends Biochem Sci 2004 29 586 592 10.1016/j.tibs.2004.09.008 15501677

[B16] Warburg O Ist die aerobe Glykolyse spezifisch für die Tumoren? Biochem Z 1929 204 482 487

[B17] Vander Heiden MG Cantley LC Thompson CB Understanding the Warburg effect: the metabolic requirements of cell proliferation Science 2009 324 1029 1033 10.1126/science.1160809 19460998 PMC2849637

[B18] Strasser-Wozak EMC Hattmannstorfer R Hála M Hartmann BL Fiegl M Geley S Kofler R Splice site mutation in the glucocorticoid receptor gene causes resistance to glucocorticoid-induced apoptosis in a human acute leukemic cell line Cancer Res 1995 55 348 353 7812967

[B19] Schmidt S Irving JA Minto L Matheson E Nicholson L Ploner A Parson W Kofler A Amort M Erdel M Hall A Kofler R Glucocorticoid resistance in two key models of acute lymphoblastic leukemia occurs at the level of the glucocorticoid receptor FASEB J 2006 20 2600 2602 10.1096/fj.06-6214fje 17077285

[B20] Löffler M Tonko M Hartmann BL Bernhard D Geley S Helmberg A Kofler R c-myc does not prevent glucocorticoid-induced apoptosis of human leukemic lymphoblasts Oncogene 1999 18 4626 4631 10.1038/sj.onc.1202820 10467407

[B21] Gossen M Freundlieb S Bender G Müller G Hillen W Bujard H Transcriptional activation by tetracyclines in mammalian cells Science 1995 268 1766 1769 10.1126/science.7792603 7792603

[B22] Helmberg A Auphan N Caelles C Karin M Glucocorticoid-induced apoptosis of human leukemic cells is caused by the repressive function of the glucocorticoid receptor EMBO J 1995 14 452 460 7859735 10.1002/j.1460-2075.1995.tb07021.x PMC398103

[B23] Fears S Chakrabarti SR Nucifora G Rowley JD Differential expression of TCL1 during pre-B-cell acute lymphoblastic leukemia progression Cancer Genet Cytogenet 2002 135 110 119 10.1016/S0165-4608(01)00655-0 12127395

[B24] Parson W Kirchebner R Mühlmann R Renner K Kofler A Schmidt S Kofler R Cancer cell line identification by short tandem repeat profiling: power and limitations FASEB J 2005 19 434 436 15637111 10.1096/fj.04-3062fje

[B25] Wu Z Irizarry RA Gentleman RC Martinez Murillo F Spencer F A model based background adjustment for oligonucleotide expression arrays Internet 2004 http://www.bepress.com/jhubiostat/paper1/

[B26] Gentleman RC Carey VJ Bates DM Bolstad B Dettling M Dudoit S Ellis B Gautier L Ge Y Gentry J Hornik K Hothorn T Huber W Iacus S Irizarry R Leisch F Li C Maechler M Rossini AJ Sawitzki G Smith C Smyth G Tierney L Yang JY Zhang J Bioconductor: open software development for computational biology and bioinformatics Genome Biol 2004 5 R80 10.1186/gb-2004-5-10-r80 15461798 PMC545600

[B27] Nicoletti I Migliorati G Pagliacci MC Grignani F Riccardi C A rapid and simple method for measuring thymocyte apoptosis by propidium iodide staining and flow cytometry J Immunol Methods 1991 139 271 279 10.1016/0022-1759(91)90198-O 1710634

[B28] Geley S Hartmann BL Hattmannstorfer R Löffler M Ausserlechner MJ Bernhard D Sgonc R Strasser-Wozak EMC Ebner M Auer B Kofler R P53-induced apoptosis in the human T-ALL cell line CCRF-CEM Oncogene 1997 15 2429 2437 10.1038/sj.onc.1201399 9395239

[B29] Gruber G Carlet M Turtscher E Meister B Irving JA Ploner C Kofler R Levels of glucocorticoid receptor and its ligand determine sensitivity and kinetics of glucocorticoid-induced leukemia apoptosis Leukemia 2009 23 820 823 10.1038/leu.2008.360 19151768

[B30] Ploner C Rainer J Niederegger H Eduardoff M Villunger A Geley S Kofler R The BCL2 rheostat in glucocorticoid-induced apoptosis of acute lymphoblastic leukemia Leukemia 2008 22 370 377 10.1038/sj.leu.2405039 18046449 PMC4950962

[B31] Mansha M Carlet M Ploner C Gruber G Wasim M Wiegers GJ Rainer J Geley S Kofler R Functional analyses of Src-like adaptor (SLA), a glucocorticoid-regulated gene in acute lymphoblastic leukemia Leuk Res 2010 34 529 534 10.1016/j.leukres.2009.06.029 19631983

[B32] Hulleman E Kazemier KM Holleman A Vanderweele DJ Rudin CM Broekhuis MJ Evans WE Pieters R den Boer ML Inhibition of glycolysis modulates prednisolone resistance in acute lymphoblastic leukemia cells Blood 2009 113 2014 2021 10.1182/blood-2008-05-157842 18978206 PMC4081395

[B33] Eberhart K Renner K Ritter I Kastenberger M Singer K Hellerbrand C Kreutz M Kofler R Oefner PJ Low doses of 2-deoxy-glucose sensitize acute lymphoblastic leukemia cells to glucocorticoid-induced apoptosis Leukemia 2009 23 2167 2170 10.1038/leu.2009.154 19657369

[B34] Nieto MA González A Gambón F Díaz-Espada F López-Rivas A Apoptosis in human thymocytes after treatment with glucocorticoids Clin Exp Immunol 1992 88 341 344 10.1111/j.1365-2249.1992.tb03084.x 1572099 PMC1554290

[B35] Brunetti M Martelli N Colasante A Piantelli M Musiani P Aiello FB Spontaneous and glucocorticoid-induced apoptosis in human mature T lymphocytes Blood 1995 86 4199 4205 7492778

