# Supplementary material for: Expression, regulation and function of phosphofructo-kinase/fructose-biphosphatases (PFKFBs) in glucocorticoid-induced apoptosis of acute lymphoblastic leukemia cells
Source: BMC Cancer. 2010 Nov 23;10:638. doi: 10.1186/1471-2407-10-638 (PMC3002928; doi:10.1186/1471-2407-10-638)
Supplement: Additional file 1 — Supplement. Supplementary information consisting of 3 Tables, 4 Figures and additional discussion section. [file 1471-2407-10-638-S1.DOC]

**Supplementary Data, Figures and Tables for**

**Expression, regulation and function of phosphofructo-kinase/fructose-biphosphatases (PFKFBs) in glucocorticoid-induced apoptosis of acute lymphoblastic leukemia cells**

Michela Carlet^1^, Kristina Janjetovic^1^, Johannes Rainer^1,2^, Stefan Schmidt^2,3^, Renate Panzer-Grümayer^4^, Georg Mann^4^, Martina Prelog^5^, Bernhard Meister^6^, Christian Ploner^1,§^ and Reinhard Kofler^1,2^

^1^Division Molecular Pathophysiology, Biocenter, Medical University of Innsbruck, 6020-Austria

^2^Tyrolean Cancer Research Institute, Innsbruck, 6020-Austria

^3^Department of Hematology and Oncology, Medical University of Innsbruck, 6020-Austria

^4^Children´s Cancer Research Institute and St.Anna Kinderspital, Vienna, Austria

^5^Department of Pediatrics I, Medical University of Innsbruck, 6020-Austria

^6^Department of Pediatrics II, Medical University of Innsbruck, 6020-Austria

§ Corresponding author

**Table of Supplemental Contents**

**Section 1: Supplementary Discussion** 3

**Section 2: Supplementary Figures and Tables**

Figure S1: GC-dependent cell death and cell cycle regulation in leukemic cell lines 4

Figure S2: Characterization of additional PFKFB2-15A overexpressing cell lines 5

Figure S3: Characterization of additional PFKFB2-15B overexpressing cell lines 6

Figure S4: Detection of endogenous PFKFB2 7

Table S1-A: *PFKFB* isoenzyme expression in primary lymphoblasts 8

Table S1-B: GC-dependent regulation of *PFKFB* isoenzymes in primary lymphoblasts 9

Table S2-A: *PFKFB* isoenzyme expression in GC sensitive and resistant

CEM-C7H2 derivatives 10

Table S2-B: *PFKFB* isoenzyme regulation in GC sensitive and resistant

CEM-C7H2 derivatives 10

Table S3: Basal expression and regulation of *PFKFB* isoenzymes - summary 12

**Section 1: Supplementary Discussion**

**Detection of endogenous PFKFB2**

Despite extensive attempts to detect endogenous PFKFB2 protein, reliable signals have not been obtained due to, as suggested by the reviewer, the sensitivity of the antibody. As shown in Figure S4 A and B, reliable detection of transgenic PFKFB2 was observed when mRNA levels reached a ΔCT (i.e., a TBP-normalized expression level) of more than ~1-2. Endogenous PFKFB2 levels prior to GC exposure in the T-ALL model cell line were, however, very low, with ΔCT levels between -6 and -7 (see Figure 1A in the manuscript) and were almost undetectable on the microarrays (see Table 1 in the manuscript). GC-induced PFKFB2 by ~7 CT steps (see Figure 1B in the manuscript), thus PFKFB2 expression reached ΔCT levels of ~0 to 1, remaining slightly below, or at the limit of, reliable detection. In addition, we noted that GCs, even though strongly inducing both PFKFB2 isoforms on the mRNA level, had some reducing effect on PFKFB2 protein levels in the transgenic cell lines (see Figure S4 C). Taken together, this explains why we failed to detect endogenous PFKFB2 proteins even after GC induction in the cell line model. Ethical reasons precluded obtaining sufficient amounts of blood from patients to perform protein analyses on the sorted lymphoblasts.

**Section 2: Supplementary Figures and Tables**

**Figure S1: GC-dependent cell death and cell cycle regulation in leukemic cell lines**


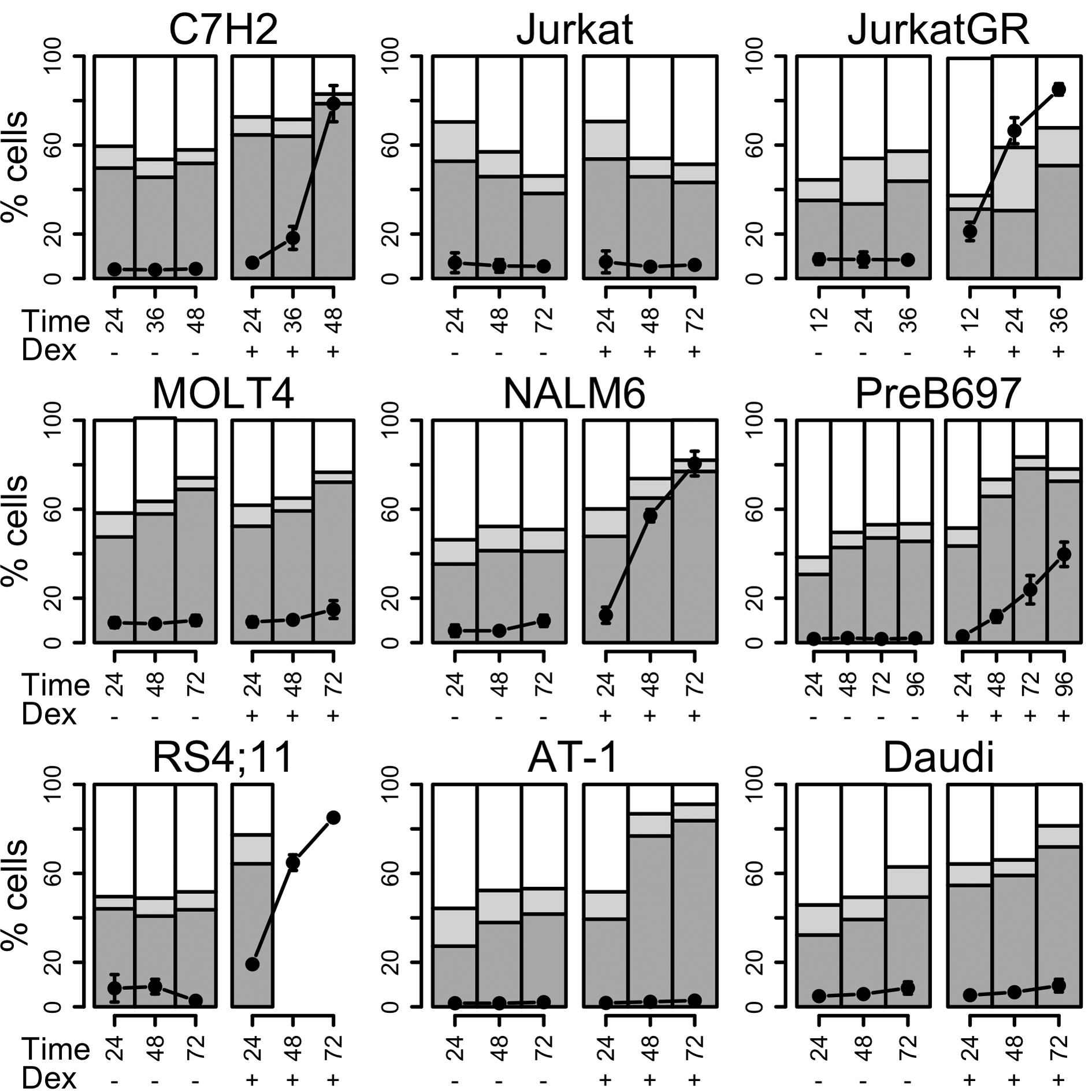


**Legend to Figure S1.** CCRF-CEM-C7H2, Jurkat (untransfected and transfected with rat GR), MOLT4, NALM6, PreB697, RS4;11, AT-1 and Daudi cells were cultured in the presence of 10^-7^M dexamethasone (Dex “+”) or 0.1% ethanol as vehicle control (Dex “-”) for the indicated time and subjected to apoptosis (•—•) or cell cycle (G1: dark gray bars; G2/M: light gray bars; S: open bars) determination using flow cytometric analysis of propidium iodide-stained nuclei. Shown are mean values ±SD of specific apoptosis (apoptosis in GC-treated samples minus apoptosis in corresponding vehicle controls) or mean values of cells in the 3 phases of the cell cycle derived from biological triplicates. In some cases, cell cycle determinations did not fulfill the required quality requirements due to high degree of apoptosis and hence were not included in the Figure. The data presented in this figure have been reported previously [18].

**Figure S2: Characterization of additional PFKFB2-15A overexpressing cell lines**

**
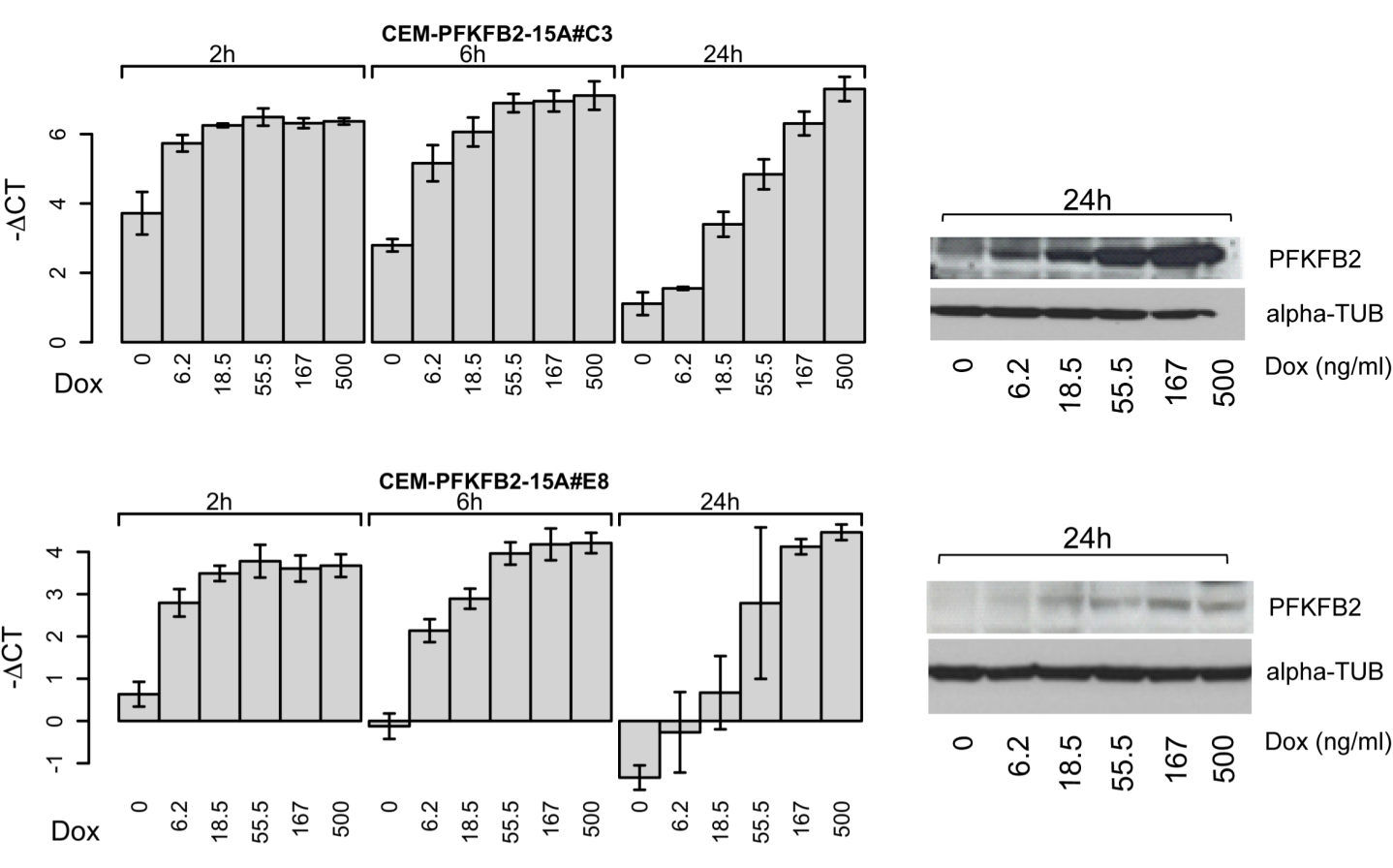
**

**Legend to Figure S2.** Additional cell lines expressing PFKFB2-15A (CEM-PFKFB2-15A #C3 and #E8) in a doxycycline-dependent manner were cultured in the presence of increasing amounts of doxycycline (Dox) and analysed for mRNA- (by quantitative RT-PCR) and protein-expression (by immunoblotting).

**Figure S3: Characterization of additional PFKFB2-15B overexpressing cell lines**

**
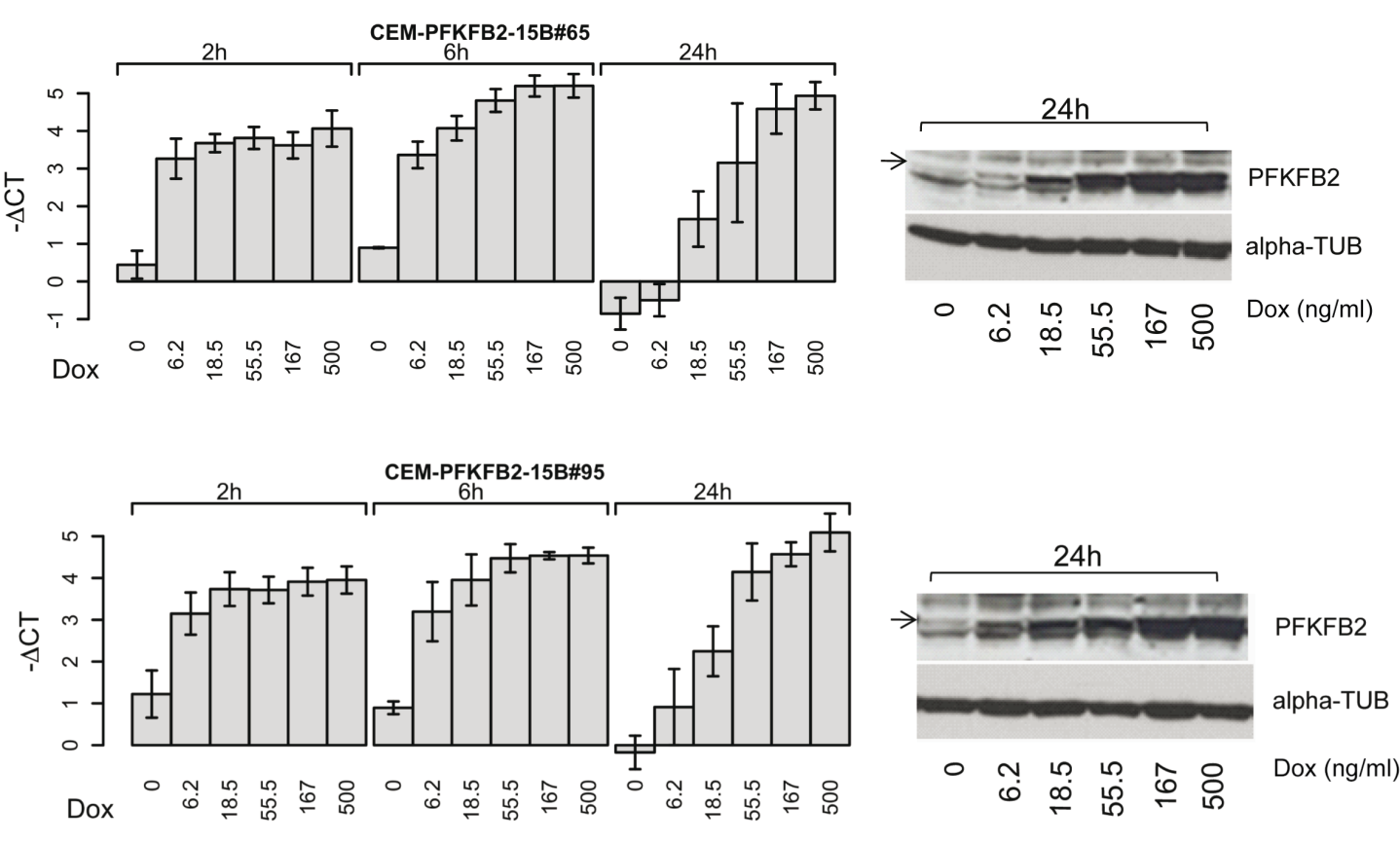
**

**Figure S3.** Additional cell lines expressing PFKFB2-15B (CEM-PFKFB2-15B #65 and #95) in a doxycycline-dependent manner were cultured in the presence of increasing amounts of doxycycline (Dox) and analysed for mRNA- (by quantitative RT-PCR) and protein-expression (by immunoblotting).

**Figure S4: Detection of endogenous PFKFB2**

**
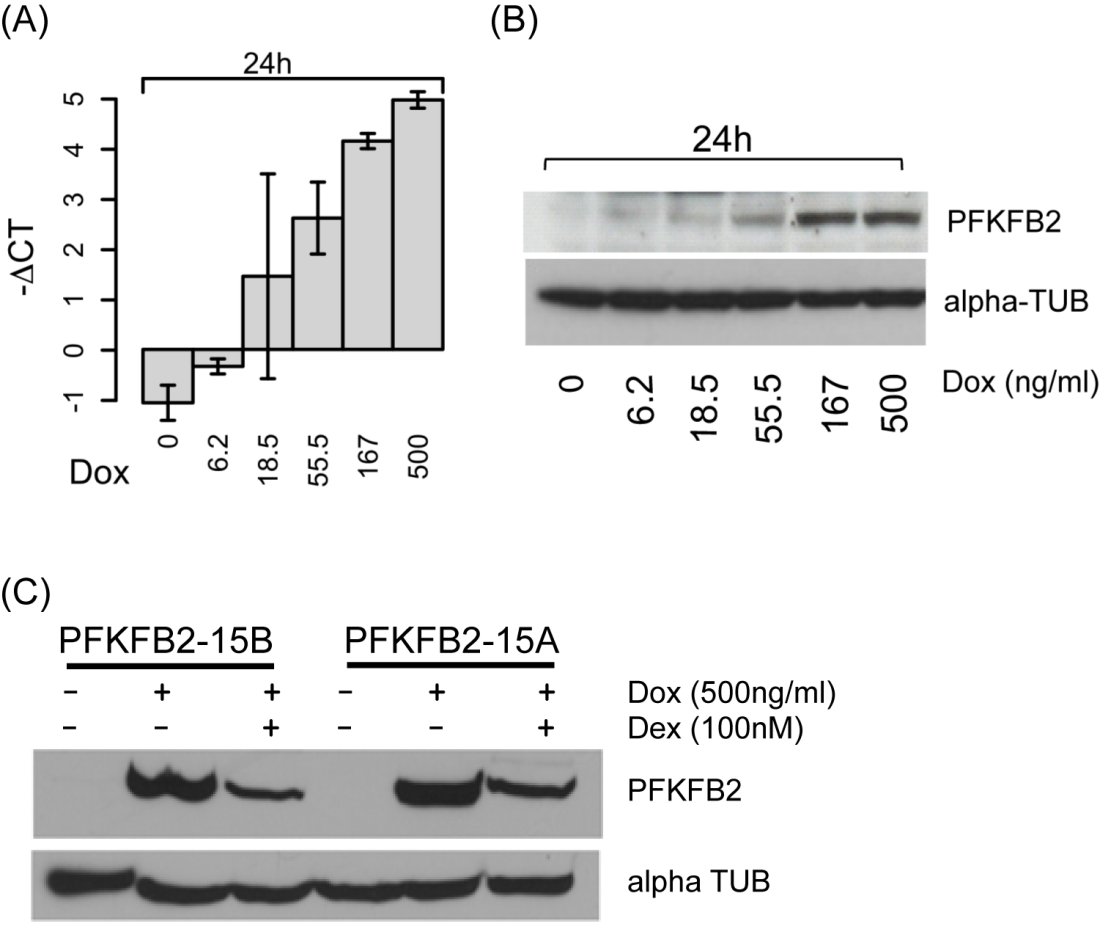
**

**Figure S4.** CEM-PFKFB2-15A#D6 cells conditionally overexpressing PFKFB2-15A were cultured in the presence of increasing amounts of doxycycline (Dox) for 24 hours and analysed for expression of PFKFB2 mRNA (A) and protein (B). (C) Immunoblot analysis of PFKFB2 in dexamethasone-treated, conditionally PFKFB2 overexpressing cells. PFKFB2-15A and -15B cell lines were cultured in the presence or absence of doxycycline for 24 hours and treated with 100 nM dexamethasone for an additional 24 hours where indicated. Although GC strongly induced both PFKFB2 isoforms on mRNA level, it also exerted some protein reducing effects in transgenic cell lines.

**Table S1-A: *PFKFB* isoenzyme expression in primary lymphoblasts of GC-treated patients**

**
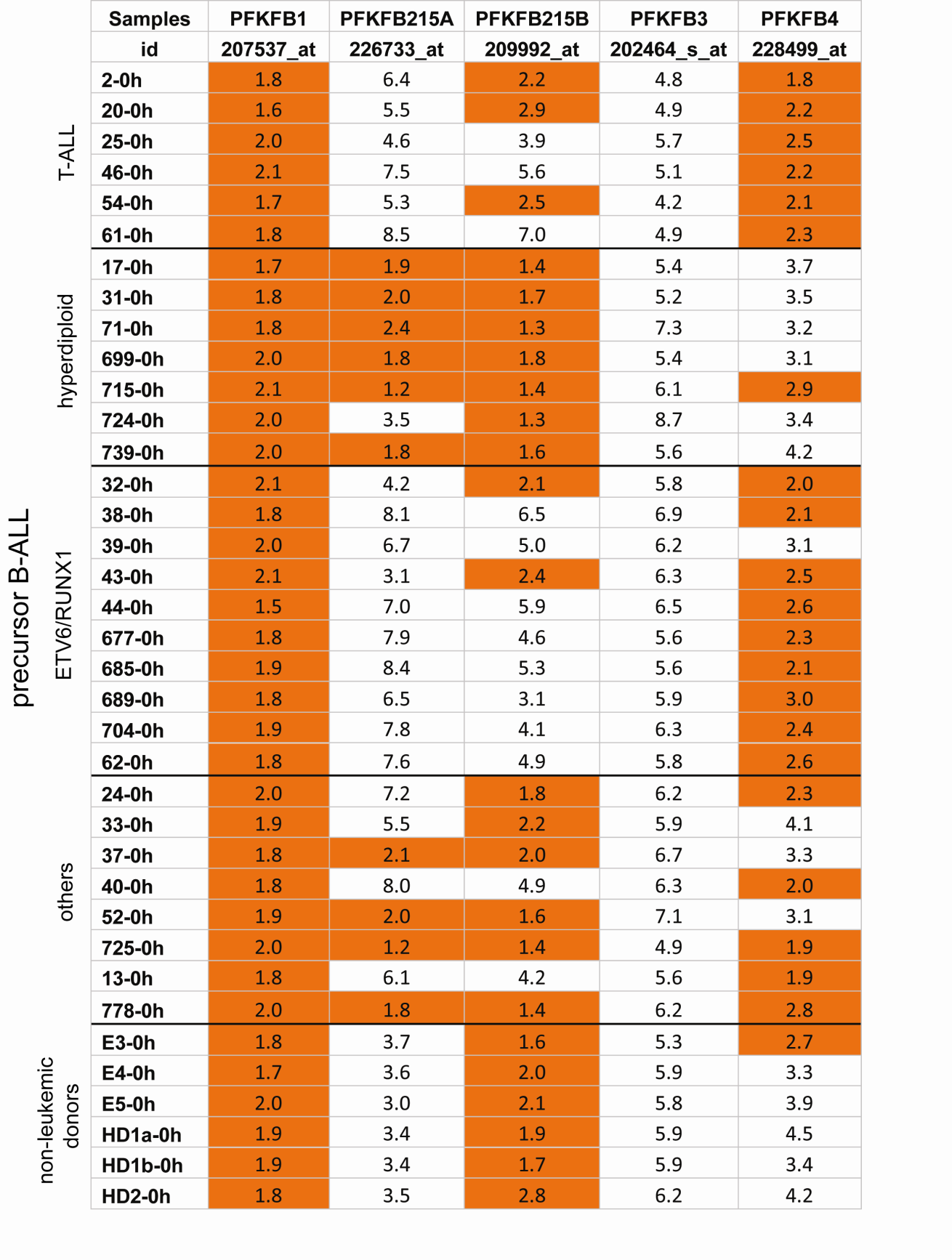
**

**Table S1-B: GC-dependent regulation of *PFKFB* isoenzymes in primary lymphoblasts**

**
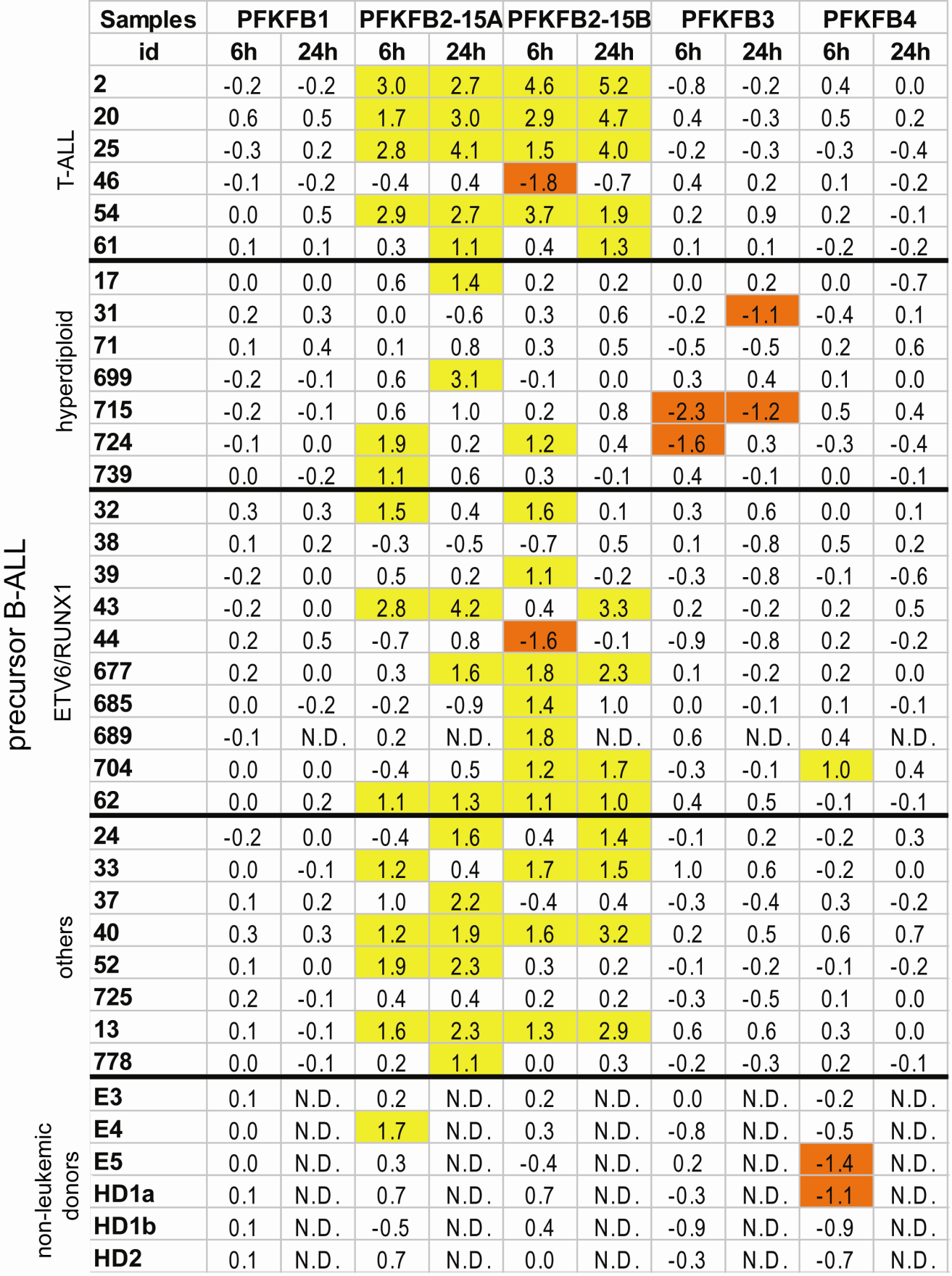
**

**Table S2-A*: PFKFB* isoenzyme expression in GC- sensitive and resistant CEM-C7H2 derivatives**

**
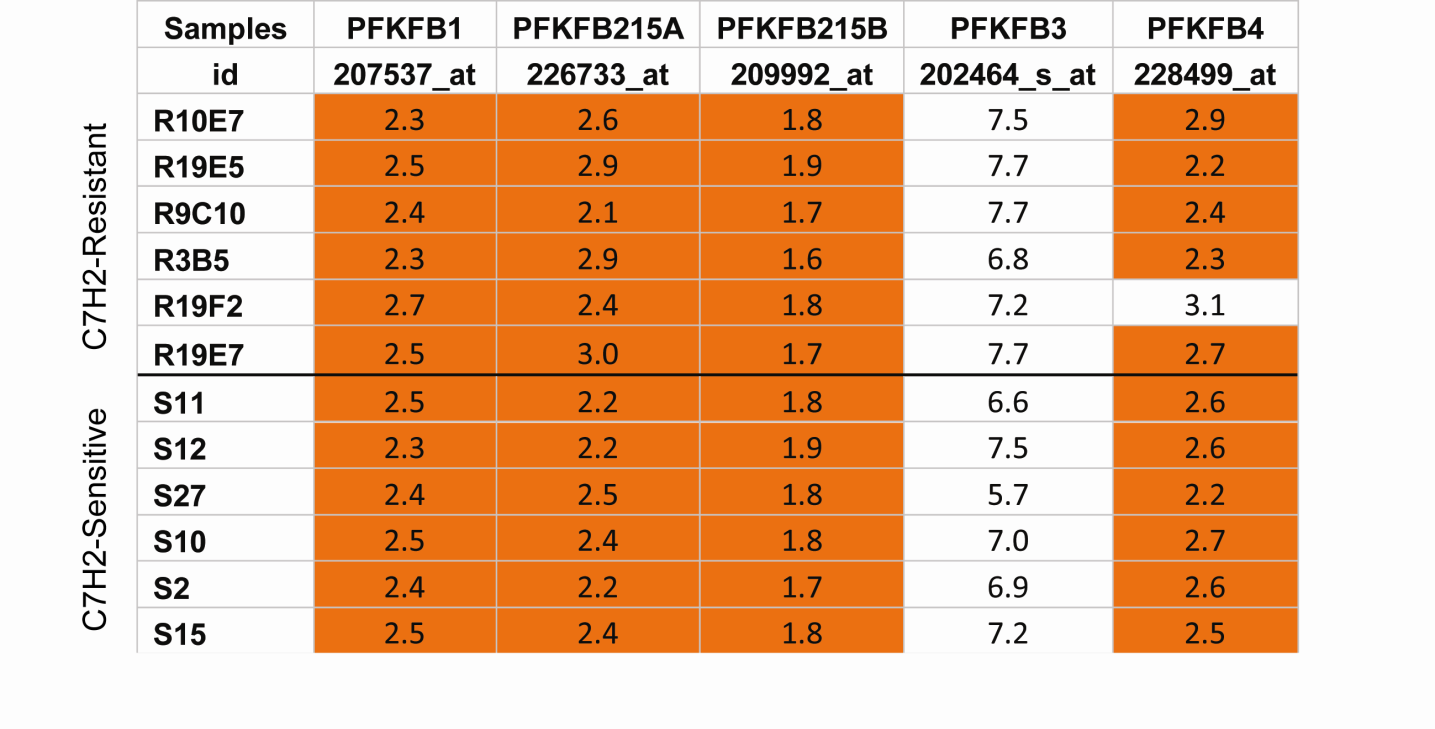
**

**Table S2-B: *PFKFB* isoenzyme regulation in GC-sensitive and resistant CEM-C7H2 derivatives**

**
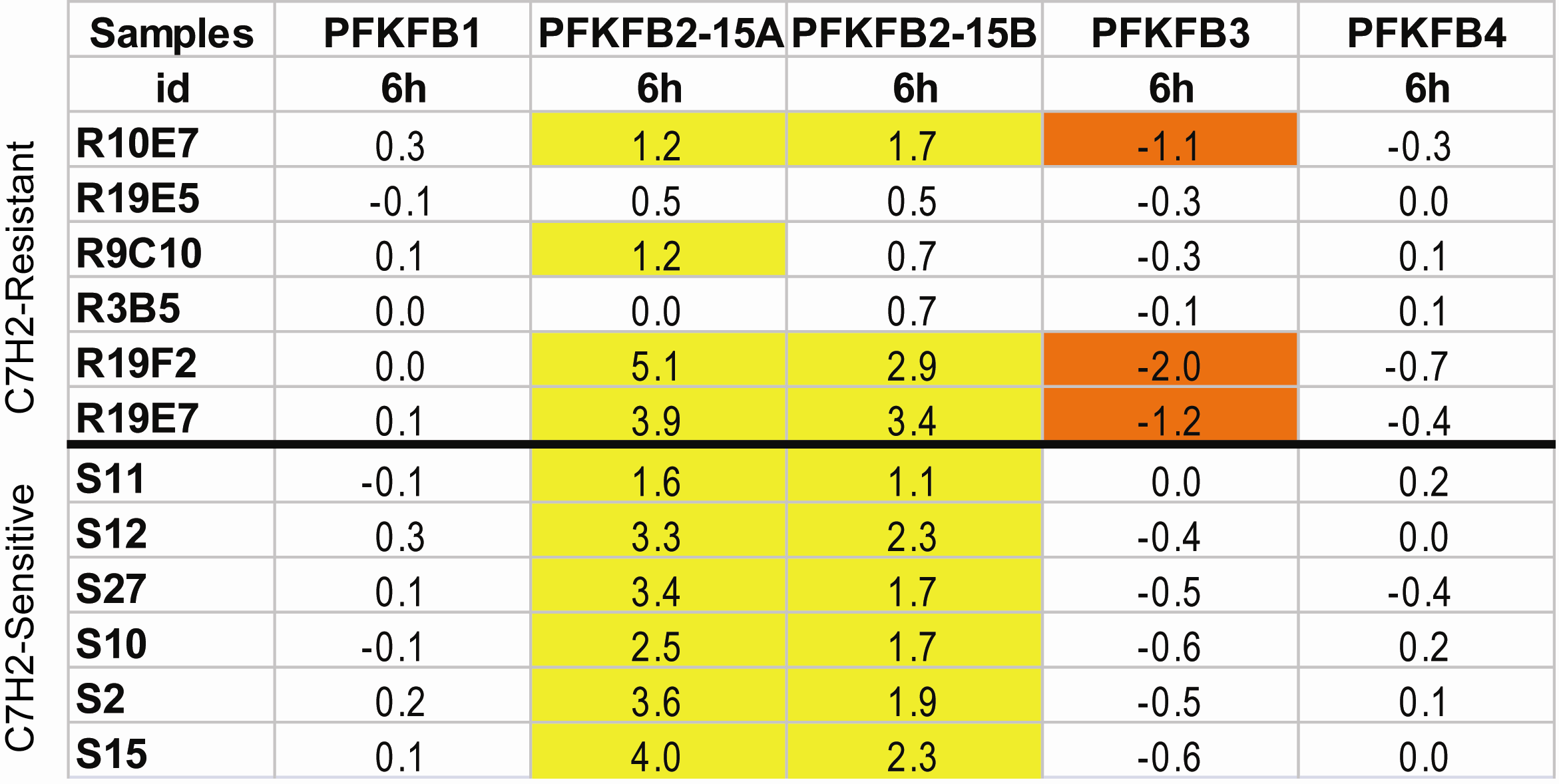
**

**Table S1 and S2.** Tables S1 (A-B) and Table S2 (A-B) summarize expression (E-value) and regulation (M-value) of the indicated genes and time points in individual ALL children, non-leukemic donors and T- ALL cell lines. A recognition number was assigned to every patient, epileptic child (E) or healthy donor (HD). HD1 was analyzed twice in 2 years (HD1a, HD1b).

Color codes:

E-values ≤3 and ≥9 are highlighted in red and yellow, respectively.

M-values ≤ -1 (equal to 2-fold down-regulation) are highlighted in red, while M-values ≥1 (equal to or more than 2-fold up-regulation) are highlighted in yellow.

Abbreviations: N.D. not determined.

**Table S3: Basal expression and regulation of *PFKFB* isoenzymes - summary**

**
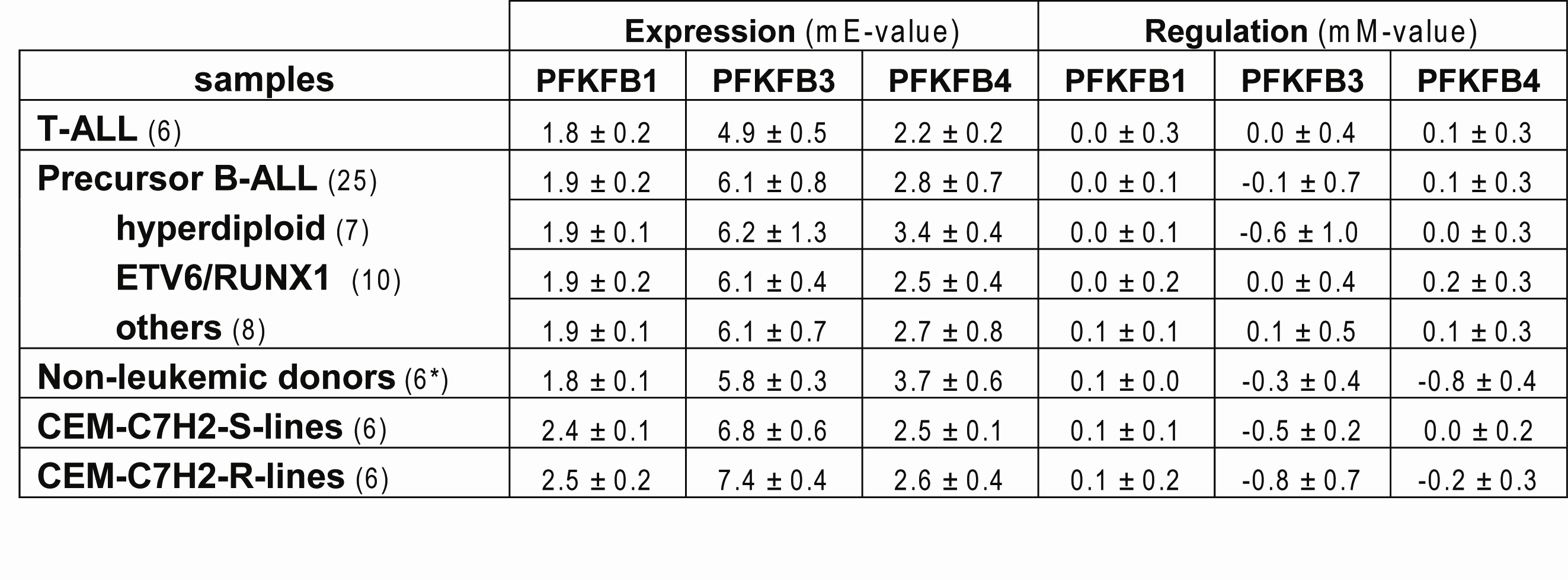
**

**Table S3.** Basal mean expression of the *PFKFB1, 3,* and *4* isoenzymes in lymphoblasts from T-ALL (n=6), precursor B-ALL (n=25) and their subgroups, peripheral blood lymphocytes from non-leukemic donors (n=6, * one healthy donor was analysed twice in 2 years), and 6 GC-sensitive and -resistant CCRF-CEM T-ALL derivatives is shown as mean expression (mE-values ± SD). GC-dependent mean regulation (mM-value±SD) after 6 hours is also shown. As a rule of thumb, E-values below 3.0 indicate no or very little expression, 4 to 8 intermediate, and 9-14 high expression. M-values of +1/-1 correspond to 2-fold up/down regulation. The slightly higher E-values in the cell lines compared to patients are probably due to the fact that cell lines and patients were normalized separately.
